# Decline in Topsoil Microbial Quotient, Fungal Abundance and C Utilization Efficiency of Rice Paddies under Heavy Metal Pollution across South China

**DOI:** 10.1371/journal.pone.0038858

**Published:** 2012-06-11

**Authors:** Yongzhuo Liu, Tong Zhou, David Crowley, Lianqing Li, Dawen Liu, Jinwei Zheng, Xinyan Yu, Genxing Pan, Qaiser Hussain, Xuhui Zhang, Jufeng Zheng

**Affiliations:** 1 Institute of Resources, Ecosystem and Environment of Agriculture, Nanjing Agricultural University, Jiangsu Nanjing, China; 2 Department of Environmental Sciences, University of California Riverside, Riverside, California, United States of America; 3 Department of Soil Science and Soil Water Conservation, Pir Mehr Ali Shah Arid Agriculture University, Rawalpindi, Pakistan; U. S. Salinity Lab, United States of America

## Abstract

Agricultural soils have been increasingly subject to heavy metal pollution worldwide. However, the impacts on soil microbial community structure and activity of field soils have been not yet well characterized. Topsoil samples were collected from heavy metal polluted (PS) and their background (BGS) fields of rice paddies in four sites across South China in 2009. Changes with metal pollution relative to the BGS in the size and community structure of soil microorganisms were examined with multiple microbiological assays of biomass carbon (MBC) and nitrogen (MBN) measurement, plate counting of culturable colonies and phospholipids fatty acids (PLFAs) analysis along with denaturing gradient gel electrophoresis (DGGE) profile of 16S rRNA and 18S rRNA gene and real-time PCR assay. In addition, a 7-day lab incubation under constantly 25°C was conducted to further track the changes in metabolic activity. While the decrease under metal pollution in MBC and MBN, as well as in culturable population size, total PLFA contents and DGGE band numbers of bacteria were not significantly and consistently seen, a significant reduction was indeed observed under metal pollution in microbial quotient, in culturable fungal population size and in ratio of fungal to bacterial PLFAs consistently across the sites by an extent ranging from 6% to 74%. Moreover, a consistently significant increase in metabolic quotient was observed by up to 68% under pollution across the sites. These observations supported a shift of microbial community with decline in its abundance, decrease in fungal proportion and thus in C utilization efficiency under pollution in the soils. In addition, ratios of microbial quotient, of fungal to bacterial and qCO_2_ are proved better indicative of heavy metal impacts on microbial community structure and activity. The potential effects of these changes on C cycling and CO_2_ production in the polluted rice paddies deserve further field studies.

## Introduction

It has been widely accepted that soil plays a key role in sustaining life of Earth ecosystems with the huge abundance of soil microbiota [1]. While crop production in agricultural lands has been increasingly threatened by heavy metal contamination [2], long term impacts of heavy metal pollution on soil microorganisms have been increasingly concerned [3], [4]. Microbial biomass has been shown sensitive to increased heavy metal concentrations in soils [3], reduction in microbial diversities and activities of soil microbial community has also been reported either in short-term lab spiked studies [5]–[7] or under long-term exposure [8]–[10] to toxic metals in soil.

Furthermore, soil bacteria and fungi are known to have different responses to heavy metal pollution [11]. Khan et al. [7] reported, by plate counting of short term incubated soil, a significant decrease in culturable bacterial colonies but no changes in fungal cells under spiked metal pollution. Likewise, in short term laboratory incubation experiments (ranging from 1 to 18 months) with spiked metals, increase in fungal proportion had been often observed using phosphorus lipid fatty acids analysis (PLFAs) assay [11]–[15]. In contrast, long term metal polluted agricultural and forest soils often exerted a reduction both in the numbers of culturable fungi cells [16] and in proportion of fungal fatty acids [17], [18].

Thus, inconsistent findings had been reported on the effects of metal pollution on soil microbial community with reference to the relative proportion of bacterial and fungal communities between short term incubation with spiked samples and long term polluted field samples and between different assays applied. Nor the potential impact of the pollution-induced microbial changes on the metabolic activity has been well addressed though reduction in total soil microbial abundance had been well recognized for the long term polluted field soils.

China’s croplands at about 2.5 Mha had been increasingly threatened by heavy metal pollution, as reported by the Ministry of Environment Protection [19]. Rice paddies had been concerned to play a key role in producing cereals for meeting the increasing food demand of China. However, extensive heavy metal pollution had been widely reported in rice paddies from the Yangtze River delta [20], [21], from the Pearl River delta [22] as well as from Jiangxi and Guangdong provinces [23], [24]. A number of studies had reported toxicities of accumulated heavy metals to the microorganisms in paddy soils under long term pollution in individual single sites of China [25]–[28]. However, an integrated understanding of the long term impacts of metal pollution on microbial abundance and community structure of rice paddy soil had yet been poorly addressed, nor of the potential changes in microbial activities well reported.

This paper reports a cross site study on the changes with long term heavy metal pollution in microbial abundance, community structure and carbon utilization efficiency of rice paddies across South China. To track any potential consistent changes in microbial abundance and activity, multiple microbiological assays were applied including MBC and MBN measurement, plate counting of culturable populations, PLFA analysis, DGGE profile and qPCR as well as soil basal respiration under laboratory incubation of field polluted samples in comparison to their background counterparts.

## Materials and Methods

### Sites

**Figure 1:**
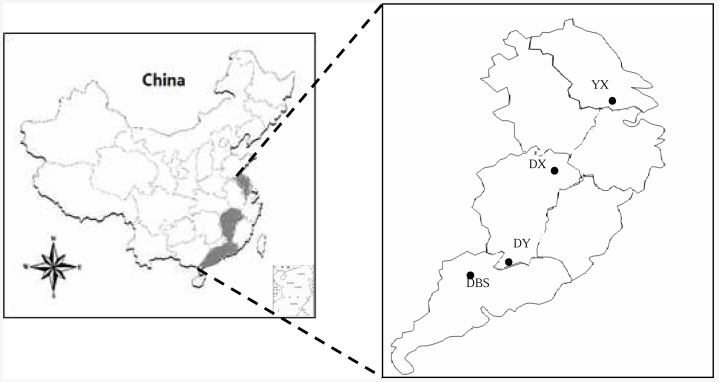
A sketch map showing the sampling sites in South China. The labels of YX, DX, DY and DBS from the north to south represent the sampling sites shown in Table 1.

In a field reconnaissance to the rice paddy region across South China in spring of 2009, a number of rice paddy areas were recognized to be polluted for more than 30 years with heavy metals due to emissions from mining and smelter activities. For a cross site study, 4 sites of rice fields were chosen including Site YX (Yixing Municipality, Jiangsu), Site DX (Dexing County,Jiangxi), Site DY (Dayu County, Jiangxi) and Site DBS (Wenyuan Municipality, Guangdong) from north to south across South China (Figure 1). For comparison, paired soil samples were collected from polluted (PS) and adjacent unpolluted fields (recognized as the background, BGS, by visits to farmers) in a single site. The studied sites are within the area controlled by subtropical monsoon climate with mean annual temperature from 18°C to 25°C, and mean annual rainfall from 1200 mm to 1450 mm from north to south, respectively. These rice paddies have been cultivated normally either with rice-rape/wheat crops or double cropping of rice in a cropping year. The basic geographic information of these sites was described in Table 1. The metal pollution status of these sites and the toxic Cd levels of rice grown in these polluted paddies were previously studied and reported [29]–[33].

**Table 1:** Location and simple pollution information of the sampled plots of the four sites studied.

Site	Location	Plot	GPS location	Pollution source and history
YX	Xushe Townshipo, Yixing Municipality, Jiangsu	BGS	31°24′10″N, 119°41′28″E	No direct pollution upwind
		PS	31°24′26″N, 119°41′36″ E	Emissions from a smelter since late 1960’s
DX	Sizhou Village, Dexing Municipality, Jiangxi	BGS	29°04′159″N, 117°43′747″E	In-access of emissions in opposite hill slope
		PS	29°04′159″N, 117°43′761″E	Waste water and particulate from a copper mine since 1950’s
DY	Fujiang Village, Dayu County, Jiangxi	BGS	25°24′200″N, 114°18′051″E	Opposite hills lope
		PS	25°24′235″N, 114°18′051″E	Waste water and solid emissions from a waste mining since 1960’s
DBS	Dabaoshan, Wengyuan County, Guangdong	BGS	24°26′702″N, 113°49′377″E	No direct pollution using upstream water for irrigation
		PS	24°27′947″N, 113°48′241″E	Intermittent waste water irrigation from a lead and zinc mining since 1960’s

BGS, background soil; PS, polluted soil.

### Topsoil Sampling and Treatment

Soil sampling was conducted before rice planting in spring of 2009 (No specific permits were required for the sampling). Three composite topsoil samples were randomly collected in depth of 0–15 cm respectively in PS and BGS plots, each of which contained 5 sub-samples collected in a “*Z*” shaped way with a distance of ∼5 m from each other and mixed thoroughly prior to shipping.

After the sampling, samples were sealed and stored in a freeze box for shipping to laboratory. In laboratory, the fresh samples were removed of soil animals and plant fragments if any, and gently crashed to pass a 2 mm sieve before being divided into four portions. The first portion was stored at 4°C for plate counting analysis and soil respiration measurement. The second and third was both stored at −20°C for PLFA and DNA extraction respectively, but the second frozen-dried before storing. The fourth, however, was air-dried and stored at room temperature for soil property measurement, of which a further portion was ground to pass a 0.15 mm sieve for determination of SOC, total nitrogen and total metal contents.

### Analysis of Soil Properties and Metal Contents

Measurements of the basic properties and metal contents of the samples were conducted following the protocols described by Lu [34]. Briefly, soil pH was measured with a glass electrode using a 1/2.5 soil/water ratio with a precision pH meter (Mettler Toledo Seveneasy, Switzerland). Soil particle content was determined with hydrometer after dispersion with 0.5 mol L^−1^ NaOH. SOC content was measured using wet digestion and oxidation with potassium dichromate. Total nitrogen was analyzed with Kjeldahl method. For total and available heavy metal content determination, the samples were digested with a mixed solution of HF/HClO_4_/HNO_3_ (10/2.5/2.5, v/v/v) and extracted with 1 mol L^−1^ HCl (1/2.5, w/v), respectively. Content of Cd was determined with graphite furnace atomic absorption spectrometry (GFAAS, SpectrAA220Z, Varian, USA), while those of Pb, Cu and Zn with flame atomic adsorption spectrophotometer (FAAS, TAS-986, China). The data of soil properties and metal contents from the above measurements are organized in Table 2 and 3 respectively.

**Table 2:** Basic physicochemical properties of the studied soil samples.

Site	Plot	SOC(g kg^−1^)	TN(g kg^−1^)	pH (H_2_O)	Clay(%)	Silt(%)	Sand (%)
YX	BGS	28.77±1.11	2.69±0.08	6.16±0.05	41.0	35.2	23.8
	PS	25.27±0.53	2.22±0.05	6.08±0.05	42.0	37.2	20.8
DX	BGS	22.79±1.58	1.58±0.05	4.87±0.05	21.0	31.2	47.8
	PS	22.25±0.35	1.96±0.07	4.10±0.06	27.0	32.2	40.8
DY	BGS	20.40±0.82	2.06±0.07	5.20±0.05	22.0	31.2	46.8
	PS	22.30±1.32	1.97±0.13	5.01±0.05	14.0	27.2	58.8
DBS	BGS	15.23±0.6	1.03±0.03	5.58±0.13	22.2	23.2	54.6
	PS	19.11±0.68	1.49±0.08	5.45±0.16	27.0	28.8	44.2

BGS, background soil; PS, polluted soil.

To evaluate the overall heavy metal pollution degree, the Nemerow pollution index [35] was followed. The index indicates an overall loading of different single polluted metal by highlighting both the maximum and average level of the determined metals. Using the Standards of Soil Environmental Quality (GB15618-1995) as soil quality assessment criteria, the Nemerow pollution index is calculated using the equation as follows:
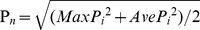
(1)Where, P*_n_* is the overall Nemerow pollution index as a sum of *n* metal elements analyzed for a soil sample, *P_i_* is a single pollution intensity index of *i*th metal element as its measured concentration (*C_i_*) divided by the concentration of reference standard (*RS*_i_). And *MaxP_i_* and *AveP_i_* is the maximum and average pollution intensity respectively of the analyzed metals in a given soil. The calculated values of Nemerow pollution index of the soils are also given in Table 3.

**Table 3:** Total and available contents of heavy metals and the Nemerow pollution index (Means ± S.D.) of the studied soils.

Sample	Total content (mg kg^−1^)				Available pool (mg kg^−1^)				Nemerow index
	Cd	Pb	Cu	Zn	Cd	Pb	Cu	Zn	
YX-B	0.45±0.01b	59.79±13.28b	42.19±0.77b	104.89±3.92b	0.21±0.03b	12.04±1.31b	7.23±0.79b	16.86±1.69b	1.20±0.04b
YX-P	6.60±0.27a	354.52±87.34a	82.56±1.90a	172.92±2.67a	4.65±0.33a	70.90±2.27a	24.88±0.22a	20.81±3.38a	16.22±0.94a
DX-B	0.48±0.14b	58.95±0.90b	640.19±2.98b	94.57±17.21b	0.10±0.08b	18.45±0.75b	191.44±24.96b	4.95±1.32b	9.45±0.03b
DX-P	1.55±0.14a	95.17±7.07a	1333.68±129.72a	163.90±15.41a	0.18±0.04a	53.18±0.53a	472.97±28.26a	13.36±2.21a	19.74±1.86a
DY-B	0.36±0.19b	68.89±7.10b	36.01±0.17b	117.14±5.58b	0.05±0.00b	13.69±0.93b	6.49±0.75b	3.75±0.33b	1.20±0.32b
DY-P	9.60±2.07a	329.76±10.60a	92.28±4.58a	368.45±16.71a	6.96±0.38a	85.78±2.67a	33.57±3.1a	120.63±6.76a	24.95±6.22a
DBS-B	0.29±0.00b	33.37±2.08b	21.87±1.02b	70.40±1.5b	0.08±0.02b	8.95±0.58b	4.84±0.19b	3.92±0.15b	0.76±0.001b
DBS-P	1.49±0.24a	133.27±6.67a	224.83±5.68a	248.48±5.87a	0.31±0.02a	37.59±1.36a	77.06±4.01a	19.98±2.23a	4.04±0.56a

Different lowercase characters in a single column indicate significant difference (*p*<0.05) between the background (B) and polluted (P) soils in a single site.

### Measurements of Soil Microbial Biomass C and N

The fumigation-extraction procedure [36] was used to determine soil MBC and MBN. The content of K_2_SO_4_-extracted C from the CHCl_3_-treated and untreated soils was determined by an automated TOC Analyzer (Shimazu, TOC-500, Japan) and a K_EC_ of 0.45 was used to convert the measured C to MBC. The total N in the extracts was measured by Kjeldahl digestion-distillation procedure and calculated as MBN with the conversion coefficient of 2.22 [37], [38].

### Plate Counting of Culturable Colonies

Plate counting of culturable microorganisms was performed basically following the procedure described by Zuberer [39]. A portion of a sample (1 g) was suspended in sterilized distilled water to form a decimal dilution up to 10^−5^. An aliquot of 50 or 100 µl of the diluted suspension of 10^−4^ or 10^−5^ was spread on a beef-protein medium plate in Petri dish, and another aliquot of 50 µl at 10^−2^ dilution on a Martin’s medium plate for counting of bacteria and fungi, respectively. Control plates of respective media without soil suspension were also included to check any possible contamination. The plates were incubated at 28°C until the visible colonies were formed. While the required incubation time was dependent of the media type, the inoculum size and the temperature of incubation [40], the proper length of incubation for clony counting was different between of fungal and of bacterial in this study. As shown in a pre-experiment respectively for fungi and bacteria, plates carrying 10 to 100 colonies of fungi and those carrying 30 to 200 colonies of bacteria [39] were counted on day 2 and on day 4 after a proper length of incubation with maximum colony size for fungal and bacterial respectively. The colony forming units (CFUs) per gram of a dry sample was then calculated. The plate incubation and counting was done in triplicates of a sample.

### Phospholipid Fatty Acid Analysis (PLFA)

#### (1) Extraction and determination

Microbial phospholipid fatty acids were extracted using the modified procedure of Bligh-Dyer [41] as described by Kehrmeyer et al. [42]. Briefly, 5 g of a frozen-dried sample was extracted with a mixture of chloroform/methanol/phosphate (1/2/0.8, v/v/v), and the lipids were separated into neutral lipids, glycol-lipids, and polar lipids [43], [44] on a pre-packed silicon acid column (Ultra-CleanTM, 500 mg/4 ml NH-2 SPE columns, Alltech, Inc.). The polar lipid fraction was trans-esterified with a mild alkali solution to recover the PLFA as methyl esters in hexane.

The PLFA obtained was dissolved with a MiDI reagent (Hexane/Methyl tert-Butyl Ether, 1/1, v/v) and analyzed with a gas chromatography (GC, HP 6890 Series, Hewlett Packard, Wilming-ton, Del.). The fatty acid 19∶0 was added as an internal standard and the different PLFAs were identified using MIDI 4.5 peak identification software (MIDI, Inc., Newark, DE, 2002). The total PLFAs concentration was expressed as nmol g^−1^ soil on a dry weight base.

#### (2) Biomarker characterization

Total microbial PLFA concentration (total PLFAs) was calculated as the sum of PLFAs in range of *C14* to *C20,* for the fatty acids with more than 20 carbon atoms were not included for presumably not of microbial origin [45]. The sum of fatty acids as bacterial PLFAs with configurations of 15∶0, 16∶0, 17∶0, i15∶0, a15∶0, i17∶0, a17∶0, i19∶0, 16∶1ω7, 16∶1w5, cy17∶0 and cy19∶0 according to Federle [46] and Frostegård et al. [12], [47], while the sum of unsaturated fatty acids was considered as fungal ones with configuration of 18∶2w6,9, 18∶1w9c and 18∶1w9t according to Federle [46] and Zelles [48]. The mole percentages of individual fatty acids to the total were also calculated.

### DNA Extraction and PCR-DGGE Assay

Total DNA was extracted with PowerSoil™ DNA Isolation Kit (Mo Bio Laboratories Inc., CA) according to the manufacturer’s protocol.

Each DNA sample was amplified with F968 and R1401 set specific for bacterial community [49] while the NS1 and Fung-GC set specific for fungal community [50]. The GC clamp described by Muyzer et al. [51] was added to the 5′ end of a primer to stabilize the melting behavior of the DNA fragments. PCR reaction was performed in an Eppendorf autothermer Cycler (Bio-Rad) using 25 µl reaction volume. The reaction mixture contained 12.5 µl Go Taq® Green Master Mix (Promega, Madison, WI), 1 µl of 10 µM of each primer, 9.5 µl of sterile ddH_2_O and 1 µl of DNA template. For DGGE analysis, PCR products were separated on a 8% (w/v) polyacrylamide gel of acrylamide/bisacrylamide (37.5/1, v/v) containing denaturing gradients of 40–60% for bacteria and 20–40% for fungi using the Bio-Rad D-Code universal mutation detection system. A 100% denaturant was defined as 8% acrylamide containing 7 M urea and 40% deionized formamide. DGGE was performed using 20 µl (for bacteria) or 11 µl (for fungi) PCR product in 1× TAE buffer at 60°C, 200 V for 5 min and then 140 V for 500 min for bacteria and 100 V for 540 min for fungi respectively. Gels were stained with silver staining [52] and then photographed with Gel Doc-2000 Image Analysis System (Bio-Rad, USA). Digitized DGGE images were analyzed with Quantity One image analysis software (Version 4.0, Bio-Rad, USA). This software identifies the bands occupying the same position in different lanes of the gel. In this approach, a band of DNA was detected greater than 1% of the total lane intensity.

### Real-time PCR (qPCR) Assay

Copy numbers of the bacterial 16S rRNA gene and the fungal internal transcribed spacer (ITS) rRNA gene of all the samples were determined in triplicates using an iCycler IQ5 Thermocycler (Bio-Rad, Hercules, CA). The quantification was based on the fluorescent dye SYBR-green one, which was bound to double stranded DNA during PCR amplification. The primers and the thermal cycling conditions used were basically following those described by Fierer et al. [53]. The DNA concentration of all samples was measured at 260 nm using a UV Spectrophotometer (Bio Photometer, Eppendorf, Germany) and then adjusted to a concentration of 15 ng µl^−1^. Each reaction was performed in a 25 µl volume containing 15 ng of DNA, 1 µl of 10 µM of each primer and 12.5 µl of SYBR premix EX Taq TM (Takara Shuzo, Shinga, Japan). Melting curve analysis of the PCR products was conducted following each assay to confirm that the fluorescence signal originated from specific PCR products but not from primer-dimers or other artifacts. PCR products were checked for the correct size by comparison to a standardized molecular weight ladder by electrophoresis on 1.5% agarose gel.

A plasmid standard containing the target region was generated for each primer set using total DNA extracted from a sample. The amplified PCR products of the bacterial 16S rRNA gene and the fungal ITS rRNA gene were purified using PCR solution purification kit (Takara, Japan), ligated into pEASY-T3 cloning vector (Promega, Madison,WI) and cloned into *Escherichia coli* DH5α. Clones containing correct inserts were chosen as the standards for qPCR. Plasmid DNA was isolated using plasmid extraction kit (Takara, Japan) and its concentration was determined with the spectrophotometer as mentioned above. As the sizes of the vector and PCR insert were known, the copies of the 16S rRNA gene and the ITS rRNA gene were directly calculated from the concentration of extracted plasmid DNA. Standard curves were generated using triplicate 10-fold dilutions of plasmid DNA ranging from 3.72×10^2^ to 3.72×10^8^ copies for the bacterial 16S rRNA gene, and 1.09×10^2^ to 1.09×10^8^ copies of template for the ITS rRNA gene per assay. High amplification efficiencies of 111% were obtained for the bacterial 16S rRNA gene and 95.3% for fungal ITS rRNA gene quantification, with a R^2^ value and a slope of 0.992 and of −3.08, of 0.995 and of −3.44, respectively. Thus, a relative fungal-to-bacterial ratio was calculated as the ratio of copy number of all the fungi species to those of bacteria species as amplified with the qPCR assay [53].

### Soil Basal Respiration (BR)

A sample was moistened to 60% of the water holding capacity (WHC) and conditioned at 25°C for 7 d under aerobic conditions to allow microbial activity stabilized before measurement. Basal respiration (CO_2_ evolution) was measured by incubating a sample equivalent to 20 g dry weight at 25°C in a 120 mL airtight jar for 7 days. The moisture of the sample was sustained at 60% of WHC consistently throughout the incubation. A gas sample from the head space of the jar was collected respectively at 2, 6, 12, 24, 36, 48, 60, 72, 96, 120 and 168 h after incubation, and the CO_2_ concentration was analyzed by Gas Chromatography (Agilent 4890D, USA) equipped with a stainless steel column (Porapak Q) (80/100 mesh) and a flame-ionization detector (FID) [54]. The basal respiration rate was expressed either on the dry soil base or on the base of soil organic carbon content.

### Data Processing and Statistical Analysis

The data presentation and treatment was processed with Microsoft Excel 2003, and the results were expressed as Means ± S.D. A paired t-test was used to check the differences between the polluted (PS) and background (BGS) samples in a single site with a significance defined at *p*<0.05. Principal component analysis (PCA) of DGGE profiles was conducted using the SPSS 16.0 statistical package for Windows to elucidate the microbial community structures based on relative band intensity and positions.

## Results

### Soil Heavy Metal Pollution

As listed in Table 2, there was no considerable difference in the basic properties between PS and BGS in a single site. However, there were consistent differences in contents of total and available Cd, Pb, Cu and Zn between the polluted and background soils across the sites though the contents of a single heavy metal element varied with sites (Table 3). As shown by the values of Nemerow pollution index (ranging from 4.0 to 25.0) of the polluted soil from a single site, the degree at which the soil was polluted with heavy metals was in an order of DY > DX > YX > DBS.

### Soil Microbial Biomass C and N, and Microbial Quotient

As shown in Table 4, soil MBC and MBN ranged from 91.2 mg kg^−1^ to 769.2 mg kg^−1^ and from 14.8 mg kg^−1^ to 69.0 mg kg^−1^ for BGSs respectively. For PSs, however, MBC and MBN ranged from 87.8 mg kg^−1^ to 474.1 mg kg^−1^ and from 11.4 mg kg^−1^ to 58.4 mg kg^−1^ respectively. A decrease in MBC under pollution was observed at a degree ranging from 24% in YX and DX to 45% in DY though not significant in DBS. The microbial quotient, a proportion of MBC to SOC, ranged from 0.6% to 3.8% and from 0.5% to 1.9% for BGSs and PSs respectively. This clearly indicated a consistently significant reduction in microbial abundance relative to SOC under metal pollution at a degree of 13% to 50% though varying with sites.

**Table 4:** Soil microbial biomass C, N and microbial quotient, as well as basal respiration and metabolic quotient of the studied soils (Means ± S.D.).

Sample	SMBC(mg kg^−1^)	SMBN(mg kg^−1^)	Microbial quotient (%)	Basal respiration(mg CO_2_–C kg^−1^ soil)	Metabolic quotient(mg CO_2_–C g^−1^ MBC h^−1^)
YX-B	623.2±35.2a	40.26±1.87a	2.17±0.03a	37.10±1.17a	0.35±0.01b
YX-P	474.1±15.6b	37.01±2.49a	1.88±0.03b	30.02±2.46b	0.40±0.01a
DX-B	238.59±21.35a	31.61±3.23a	1.05±0.09a	22.64±1.29a	0.56±0.03b
DX-P	181.07±12.11b	11.51±0.64b	0.81±0.05b	22.61±1.08a	0.74±0.04a
DY-B	769.2±25.01a	69.02±3.88a	3.77±0.12a	24.29±2.52a	0.19±0.02b
DY-P	419.47±45.00b	58.35±3.68b	1.88±0.20b	22.30±5.27a	0.32±0.08a
DBS-B	91.56±10.58a	14.78±2.89a	0.60±0.07a	19.56±0.76a	1.25±0.02b
DBS-P	87.78±10.07a	11.41±3.15a	0.46±0.06b	17.41±1.67a	1.34±0.03a

Different lowercase characters indicate significant difference (*p*<0.05) between background (B) and polluted (P) soils in a single site.

### Culturable Microbial Population Abundance

**Figure 2:**
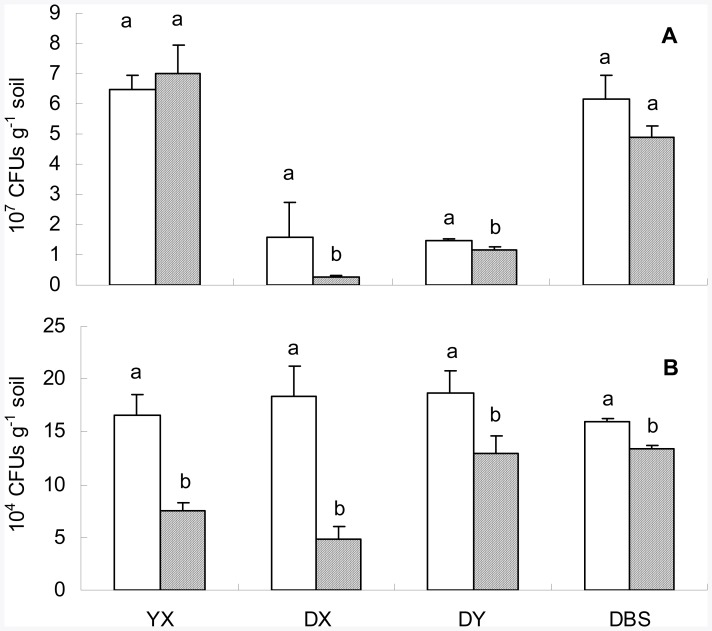
Culturable population of bacteria (A) and of fungi (B) of the soils studied. Blank, background soil; Shaded, polluted soil. Different lowercase characters indicate significant difference (*p*<0.05) between the background and polluted soils in a single site.

Data of culturable colony counting from the plate incubation experiment is shown in Figure 2. Population size of culturable bacteria ranged from 1.5×10^7^ CFUs g^−1^ in DY to 6.5×10^7^ CFUs g^−1^ in YX for BGSs, and from 0.3×10^7^ CFUs g^−1^ in DX to 7.0×10^7^ CFUs g^−1^ in YX for PSs. Meanwhile, that of culturable fungi from 16.0×10^4^ CFUs g^−1^ in DBS to 18.7×10^4^ CFUs g^−1^ in DY for BGSs, and from 4.8×10^4^ CFUs g^−1^ in DX to 13.4×10^4^ CFUs g^−1^ in DBS for PSs. Here is clearly seen a consistent reduction by 16% to 74% under pollution in the size of culturable fungal population across the four sites despite of an inconsistent change in that of culturable bacteria. Nevertheless, the change with pollution in the ratio of fungal to bacterial culturable colonies was not consistently seen across the sites.

### Extractable PLFAs

Data of PLFA analysis is presented in Figure S1. There were totally 36, 35, 30 and 25 single PLFAs detected in BGS and PS from YX, DY, DX and DBS sites respectively, of which 16 fatty acids were identified common across the sites. Furthermore, the fatty acid 18∶4 w3c was detected in all the PSs, while only in the BGS of DY. Whereas, the 18∶1 w5c was not detected in the PSs, except in that of YX with a much lower content of PLFA extracted. The concentration of fatty acid 18∶1 w9t (one of fungal biomarkers) was shown to reduce in all PSs compared to in BGSs.

**Figure 3:**
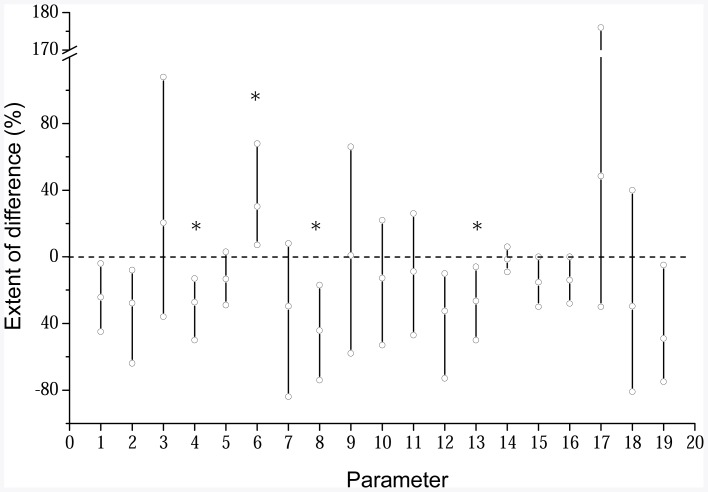
Overall relative changes in analyzed microbial parameters under metal pollution to the background soils. The upper, middle and lower hollow circle in a vertical bar represents the maximum, average and minimum changes under pollution to the background in a single parameter. 1 and 2, Microbial biomass carbon and nitrogen; 3 and 4, Ratio of microbial biomass C and N to soil organic carbon; 5, Basal respiration; 6, Metabolic quotient (qCO_2_); 7 and 8, Bacterial and fungal CFUs; 9, Ratio of fungal to bacterial CFUs; 10, 11 and 12, Total, bacterial and fungal PLFAs; 13, Ratio of fungal to bacterial PLFAs; 14 and 15, Bacterial and fungal DGGE band numbers; 16, Ratio of fungal to bacterial band numbers; 17 and 18, Bacterial and fungal gene copies; 19, Ratio of fungal to bacterial gene copies.

The concentrations of PLFAs of the soils studied are organized in Table 5. The extracted total, bacterial and fungal PLFAs of the BGSs ranged from 11.2 nmol g^−1^ in DBS to 49.1 nmol g^−1^ in DY; from 6.4 nmol g^−1^ in DBS to 24.3 nmol g^−1^ in YX, and from 2.1 nmol g^−1^ in DBS to 13.8 nmol g^−1^ in DY respectively. Comparatively, these PLFAs showed narrow ranges in PSs. The concentration of fungal PLFAs was found reduced under pollution by 14% to 73% consistently across the sites while a significant decrease in both total and bacterial PLFAs under pollution was detected only in sites of YX and DY. In turn, the calculated ratio of fungal to bacterial PLFAs was significantly lower by 6% to 50% in PSs than in BGSs across the sites (Figure 3).

**Table 5:** Concentrations (nmol g^−1^) of total, bacterial, fungal PLFAs and the fungal-to-bacterial PLFA ratio (Means ± S.D.) of the studied soils.

Sample	Total PLFAs (nmol g^−1^)	Bacterial PLFAs (nmol g^−1^)	Fungal PLFAs (nmol g^−1^)	Ratio of fungal to bacterial
YX-B	46.79±3.06a	24.32±1.62a	8.59±0.66a	0.35±0.02a
YX-P	32.63±3.49b	17.47±1.68b	5.78±0.62b	0.33±0.01b
DX-B	18.65±2.04a	9.93±1.10a	4.02±0.32a	0.40±0.03a
DX-P	20.55±0.92a	11.28±0.46a	3.47±0.26b	0.31±0.02b
DY-B	49.06±6.10a	23.18±2.93a	13.81±1.11a	0.60±0.04a
DY-P	22.98±7.27b	12.26±0.37b	3.72±0.28b	0.30±0.03b
DBS-B	11.23±1.09a	6.37±0.47b	2.12±0.29a	0.33±0.04a
DBS-P	13.67±1.79a	8.01±0.65a	1.91±0.36a	0.24±0.03b

Different lowercase characters in a single column indicate significant difference (*p*<0.05) between the background (B) and polluted (P) soils in a single site.

### Bacterial and Fungal DGGE Bands and Gene Copy Numbers

Bacterial and fungal DGGE band patterns both of BGSs and PSs from the four studied sites are given in Figure S2A and S2B, respectively. The data of bacterial and fungal band numbers of 16S rRNA and 18S rRNA gene retrieved from the DGGE profiles and gene copies measured by qPCR of the studied soils are organized in Table 6.

**Table 6:** Bacterial and fungal DGGE band numbers and gene copy numbers (Means ± S.D.) of the soils studied.

Sample	DGGE band number			Gene copy number		
	Bacteria	Fungi	F/B	Bacteria (×10^10^)	Fungi (×10^8^)	F/B (×100)
YX-B	30±1a	25±0a	0.84±0.02a	5.46±1.20a	14.3±4.4a	2.68±0.10a
YX-P	30±2a	25±1a	0.84±0.02a	3.80±0.44a	9.50±3.50a	2.50±0.87a
DX-B	35±1a	20±1a	0.59±0.02a	0.85±0.14b	1.35±0.29a	1.60±0.33a
DX-P	34±1a	14±1b	0.41±0.01b	1.46±0.05a	0.76±0.09b	0.52±0.07b
DY-B	32±1a	27±2a	0.85±0.06a	1.16±0.09a	4.20±1.44a	3.58±0.95a
DY-P	29±2a	20±2b	0.66±0.02b	0.88±0.08b	0.81±0.61b	0.91±0.64b
DBS-B	32±1a	22±1a	0.69±0.01a	0.21±0.16a	0.48±0.19a	2.43±0.48a
DBS-P	34±1a	21±1a	0.62±0.01b	0.58±0.10a	0.67±0.08a	1.16±0.01b

F/B represents the ratio of fungi to bacteria. Different lowercase characters in a single column indicate significant difference (*p*<0.05) between the background (B) and polluted (P) soils in a single site.

The gene copy number of bacteria and fungi ranged from 0.2×10^10^ to 5.5×10^10^ and from 0.5×10^8^ to 14.3×10^8^ for BGSs, and from 0.6×10^10^ to 3.8×10^10^ and from 0.7×10^8^ to 9.5×10^8^ for PSs respectively. Unlike that of bacteria, here the copy number of fungi was seen more or less reduced under pollution in all sites except in DBS. The calculated ratio of fungal/bacterial copy number was seen decreased under pollution slightly in YX site and significantly in the other three sites. Following a similar trend, the band numbers of bacteria from the 16S rRNA gene DGGE profile were consistently higher than those of fungi from 18S rRNA gene profile. However, there was no difference in the bacterial band numbers between PS and BGS in a single site, being 32±2 on average. Whereas, band numbers of fungi under pollution were seen unchanged in YX and more or less reduced in other three sites. As a result, the calculated ratio of fungal-to-bacterial band numbers was reduced significantly in sites of DX, DY and DBS though no change in YX. These data supported a relative reduction in gene copies and band numbers of fungi rather than of bacteria under heavy metal pollution in the rice paddies.

### Bacterial and Fungal Community Structure

**Figure 4:**
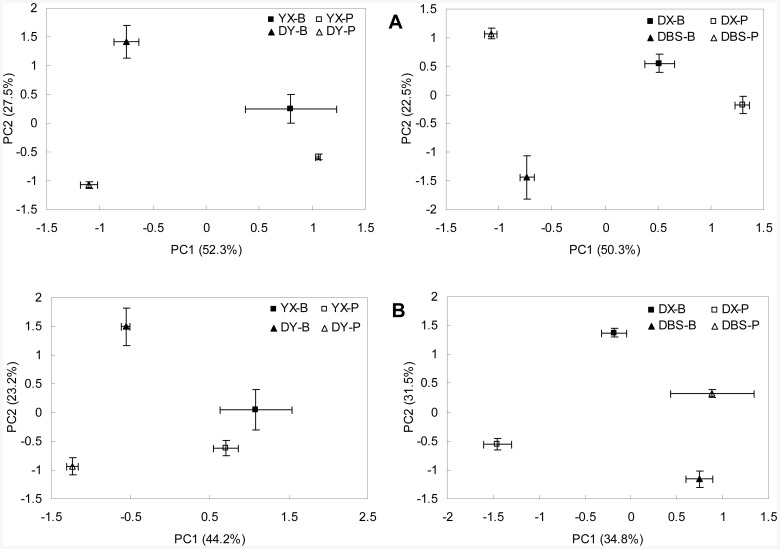
Principal component analysis of DGGE profiles of bacterial 16S rRNA (A) and fungal 18S rRNA (B) gene fragments. YX-B and YX-P, Background and polluted soil of site YX; DX-B and DX-P, Background and polluted soil of site DX; DY-B and DY-P, Background and polluted soil of site DY; DBS-B and DBS-P, Background and polluted soil of site DBS, respectively.

The principal component analysis (PCA) of DGGE profiles both of bacterial 16S rRNA and fungal 18S rRNA gene fragments are shown in Figure 4A and 4B, respectively. PCA scores of bacterial DGGE profile showed significant separation (*p*<0.5) along PC1, which accounted for around 50% of the variances, between PS and BGS in DX, DY and DBS sites. For fungal DGGE profiles, the significant divergence between PS and BGS along PC1 was found in DX and DY not in YX and DBS. Moreover, the significant divergence along PC2 between PS and BGS was found in all sites for both bacterial and fungal profiles. A shift indicated here in bacterial and fungal community structure supplemented the above microbial changes of the rice paddies with heavy metal pollution.

**Figure 5:**
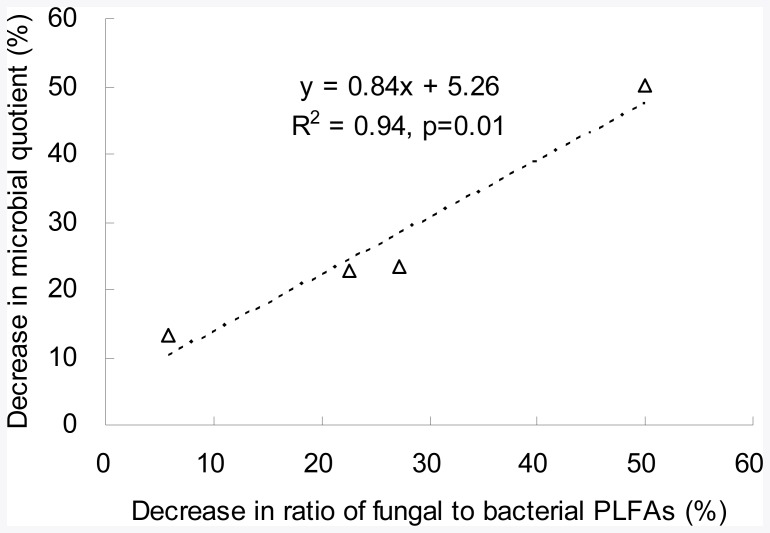
Reduction in microbial quotient (%) versus decrease in fungal-to-bacterial PLFAs ratio (%) under pollution.

**Figure 6:**
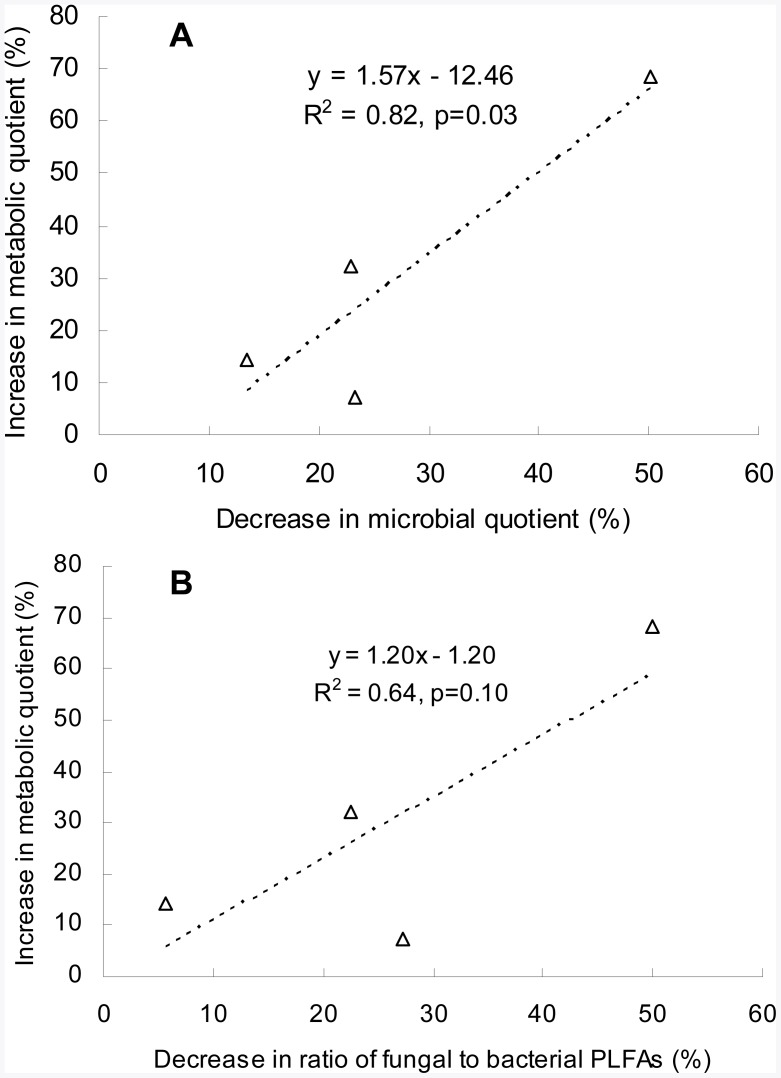
Increase in metabolic quotient (%) versus decrease in microbial quotient (%) (A) and in fungal-to-bacterial PLFAs ratio (%) (B) under pollution.

### Basal Respiration

Data of the basal respiration in the incubation course of the studied soils is shown in Figure S3. Calculated with the cumulic CO_2_ production from the whole incubation course, the basal soil respiration rates for the studied soils are given in Table 4. Ranging from 17 mg CO_2_–C kg^−1^ soil to 37 mg CO_2_–C kg^−1^ soil, total basal respiration was not seen different between BGS and PS from a single site except for YX. The basal respiration normalized on the basis of SOC in all sites was not significantly changed under pollution, ranging from 1.0 mg CO_2_–C g^−1^ SOC to 1.3 mg CO_2_–C g^−1^ SOC for BGSs and from 1.0 mg CO_2_–C g^−1^ SOC to 1.1 mg CO_2_–C g^−1^ SOC for PSs. This seemed a similar SOM feature between the BGS and PS in a single site. Whereas, the calculated metabolic quotient, the basal respiration rate on basis of MBC, ranged from 0.2 mg CO_2_–C g^−1^ MBC h^−1^ to 1.2 mg CO_2_–C g^−1^ MBC h^−1^ for BGSs, and from 0.3 mg CO_2_–C g^−1^ MBC h^−1^ to 1.3 mg CO_2_–C g^−1^ MBC h^−1^ for PSs (Table 4). There was a clear cross-site trend of increase in the metabolic quotient under pollution at a degree of 7% to 68% (Figure 3).

## Discussion

### Consistent Changes in Microbial Community with Heavy Metal Pollution in the Rice Paddies

In literature, either decrease in the size of the microbial biomass [28], [55], or no change in MBC [56], or a reduction in culturable bacterial and fungal population size [16], or a decrease in fungal fatty acids [57], as well as a shifted fungal and bacterial communities [58] had been reported for long term metal polluted croplands. A decrease in culturable fungal population was also seen in the study of Nordgren et al. [59] who found the isolated colonies of culturable fungi reduced in a soil close to a brass mill under coniferous forest. Decrease in fungal to bacterial PLFA ratio was detected in an uncultivated soil under metal pollution from acid mining spill [60], and in forest soils along a metal pollution gradient [17].

However, there had been not yet clear and sound understanding of a well established and coherent change in soil microbial community structure and abundance with heavy metal pollution in fields across land use types of soil and metal pollution status. This could be partially because of the varying effects of heavy metal pollution on soil microbial community with pollution status. The changes in microbial populations or processes under field pollution differed from under spiked treatment [61], [62], and those with short term pollution [11]–[13] differed from with long term pollution [17], [57]–[59]. In addition, the effect could be also subject to change with metal toxicity to microorganisms as affected by soil reaction and pollution history of the sites studied [3]. Co-existence of multiple metal elements with varying eco- toxicities to targeted microbial communities [63], [64] would have influences in the overall changes in soil microbial community with metal pollution across sites.

An overall comparison of the changes relative to the background in all the analyzed parameters conducted in this study is shown in Figure 3. Among the total 19 parameters analyzed, 3 exerted a consistent and significant decrease across sites under the pollution, including microbial quotient, fungal CFUs and ratio of fungal to bacterial PLFAs. Meanwhile, 1 parameter of the metabolic quotient (qCO_2_) showed a consistent and significant increase under the pollution across the sites. The other parameters such as soil MBC and MBN, fungal PLFAs, fungal DGGE band numbers and ratios of fungal to bacterial band numbers and gene copies showed relatively week trend of decrease under pollution though the others varied widely with sites. Here it is clearly that the parameters showing consistent changes across the sites are mostly those of or associated with fungal abundance and proportion.

Rice paddy had been concerned as a unique anthropogenic soil type developed with a particular land use with similar agricultural management for rice production [65]. There had been a number of studies on soil microbial community changes in polluted soil from single sites. These studies reported a general trend of decline in MBC and the microbial quotient in rice paddies under metal pollution of China [25], [26]. Using PLFA assay, Yan et al. [26] could indicate a significant decrease in fungal to bacterial PLFA ratio under heavy metal pollution in a rice paddy from the Tai Lake region of China. This study, by using multiple microbial biological assays for long term polluted soils across sites, portrayed a consistent changes characterized mainly by the reduction in fungal population size and the relative proportion in the metal polluted rice paddies across South China.

### Change in Metabolic Activity and the Coherence to Microbial Community Change Under Pollution

It has been well known that microbial community physiologically links to ecosystem C and N cycling and balance [66], [67]. Changes in microbial community composition had been well documented to modify ecosystem processes through their changes in physiological processes and thus could exert an important role in terrestrial ecosystems functioning [68], [69]. Changes in fungal abundance and species richness due to disturbances would affect the decomposition of soil organic matter [70]. Six et al. [71] noted that fungal-dominated soils had slow C turnover rates. The role of such a shift of microbial community composition was also argued in a recent work by Zhou et al. [72] as one of the primary feedback mechanisms through which microorganisms regulated soil carbon dynamics. Changes in C utilization efficiency by soil microorganisms under metal pollution had been already proposed as a well measure of microbial response to disturbance and had been considered a sensitive ecophysiological indicator of heavy metal stressed soil [73].

As the soil bacterial and fungal community structure shifted (Figure 4) and the fungal abundance and band numbers showed a reduced tendency under pollution in the present study, it could have further impacts on SOC turnover and the C utilization efficiency of the rice paddies. Here, the reduction in microbial quotient under pollution was significantly related to the decreased fungal-to-bacterial PLFAs ratio (Figure 5). And the increase in qCO_2_ was significantly (*p*<0.05) correlated with the reduction in microbial quotient (Figure 6A), and moderately significantly (*p* = 0.10) correlated with the decreased fungal-to-bacterial PLFAs ratio (Figure 6B) under pollution. Thus, a significant increase in microbial metabolic quotient, an indicator of C utilization efficiency of soil, together with the decline in microbial quotient and fungal to bacterial ration of extracted PLFAs, implicated a potential impact on soil organic carbon protection and CO_2_ release under heavy metal pollution. The finding here was in agreement with the observation by Chander and Brookes [74] who reported a lower efficiency of C utilization and higher rates of CO_2_ evolution from a high-metal soil than from a low-metal one after amending with maize or glucose. Such fact could also be seen in the work by Li [75] who reported significant increases by over 20% both in basal respiration under lab incubation and field CO_2_ evolution from a rice paddy under heavy metal pollution when compared to the non-polluted counterpart. With the same paddy soil as by Li [75], Zhang et al. [76] observed a destruction of water stable coarse micro-aggregates, which had been believed as a physical protection of soil organic matter in soils [77]. Decreased C utilization efficiency was also reported by Zhou et al. [78] who conducted a laboratory incubation and CO_2_ evolution monitoring with different soils from long-term polluted fields in 6 sites across South China though they failed to show a consistent trend of change in qCO_2_ with heavy metal loadings. In contrast, increase in fungal abundance and the fungal to bacterial ratio from culturable colony analysis was found in our previous work in good correlation with the soil organic matter accumulation of rice paddy soils from long term experiment sites across South China [79]. Therefore, a consistently higher metabolic quotient by an extent ranging from 7% to 68% over the polluted ones found in this study could indicate a strong potential impact through the shift with reduction in fungal abundance and proportion of heavy metal pollution on C dynamics and CO_2_ production from the rice paddies. Of course, the consequence of such impacts and the mechanism behind in relation to the microbial shift deserves further field monitoring studies.

### Intercomparison of Different Assays for Interpreting Heavy Metal Impact on Microbial Community Shift

Many methods had been available for characterizing environmental stress on soil microbial community structure and activity. Plate counting of culturable microorganisms had been shown to be an effective method for depicting heavy metal pollution [80], [81]. PLFA analysis, however, provided phenotypic information of the active microbial community composition [12], [82] as well as the microbial biomass [83], being used extensively to address environmental stresses [84], [85]. More recently, molecular techniques of PCR-DGGE had been increasing employed for monitoring microbial community and diversity changes under environmental stresses [86]. Furthermore, qPCR in combination with PCR-DGGE had been proven as a molecular fingerprinting measure to track the gross differences and changes in microbial population size and structure with a certain kind of stresses in soils [87].

In this study, multiple microbial biological assays ranging from microbial biomass C and N, to molecular fingerprinting techniques were used. Measurements of microbial quotient, fungal population size and the ratio of fungal to bacterial PLFAs yielded consistently great difference between the polluted and background soils across sites by up to over 50%. In contrast, molecular fingerprinting yielded less consistent and significant difference between the polluted and the background soils (Figure 3). Again, neither the absolute contents of microbial biomass C and N, nor the abundance and DGGE bands of bacteria with each of the methods employed succeeded to characterize the microbial changes with metal pollution in this study. Whereas, a ratio of fungal to bacterial from almost all the measurements together with the qCO_2_ indicator proved valid to portray the changes in microbial community shift under heavy metal pollution. Therefore, combining the traditional culture-dependent method and the advanced PLFA fingerprinting approach could be employed as a tool for well identifying the changes in soil microbial communities characterized by a reduction in fungal abundance and proportion in soils under heavy metal pollution despite of the inherent bias of each method [88]. It is the authors’ suggestion that use of multiple biological assays by the means of comparison between the polluted and background soils across sites could be a sound methodology for studying microbial community structure shift under environmental stress in croplands.

### Conclusions

The present study, by using multiple microbial biological assays including measurement of microbial carbon and nitrogen, plate counting of culturable colonies, PLFA analysis, DGGE profile and qPCR along with soil basal respiration measurement under lab incubation, revealed a consistent change in soil microbial community and metabolic activity under heavy metal pollution of rice paddies across South China. These changes could be characterized by a decline in abundance of overall microbial community and specifically of fungi, in fungal to bacterial ratio as well as an increase in metabolic quotient. Thus, heavy metal pollution could exert significant impact on soil biogeochemical process related to C metabolism and CO_2_ evolution from rice paddies. The measurements of soil microbial abundance, population size of culturable colonies as well as extractable PLFAs proved valid to portray the above-mentioned changes under pollution and ratio parameters of microbial quotient, of fungal to bacterial and qCO_2_ are better indicators for characterizing heavy metal impacts on soil microbial community structure and activity shift with heavy metal pollution in soils. However, the potential impact of heavy metal pollution on soil C cycling would be a critical issue for environmental studies in the near future as the area of heavy metal pollution of rice paddies had been extending in China.

## Supporting Information

Figure S1 The PLFA profiles of the soils studied. Blank, background soil; Shaded, polluted soil.
(DOC)

Figure S2 DGGE profiles of amplified bacterial 16S rRNA (A) and fungal 18S rRNA (B) gene fragments. YX-B and YX-P, Background and polluted soil of site YX; DX-B and DX-P, Background and polluted soil of site DX; DY-B and DY-P, Background and polluted soil of site DY; DBS-B and DBS-P, Background and polluted soil of site DBS, respectively. M, Marker. White asterisks highlight bands which intensified or only existed in the profiles of background soils; whereas, white rectangles highlight those which intensified or only existed in the polluted counterparts in a single site.
(DOC)

Figure S3 Soil basal respiration course under lab incubation at constantly 25°C of the soils studied. Blank, background soil; Shaded, polluted soil.
(DOC)
