# Supplementary material for: Decline in Topsoil Microbial Quotient, Fungal Abundance and C Utilization Efficiency of Rice Paddies under Heavy Metal Pollution across South China
Source: PLoS One. 2012 Jun 11;7(6):e38858. doi: 10.1371/journal.pone.0038858 (PMC3372496; doi:10.1371/journal.pone.0038858)
Supplement: Figure S1 — The PLFA profiles of the soils studied. Blank, background soil; Shaded, polluted soil. (DOC) [file pone.0038858.s001.doc]

Fatty acids

Mol % of total fatty acids

**YX**

**DX**

**DY**

**DBS**

**Figure S1**
