# Supplementary figures and images for: Decline in Topsoil Microbial Quotient, Fungal Abundance and C Utilization Efficiency of Rice Paddies under Heavy Metal Pollution across South China
Source: PLoS One. 2012 Jun 11;7(6):e38858. doi: 10.1371/journal.pone.0038858 (PMC3372496; doi:10.1371/journal.pone.0038858)

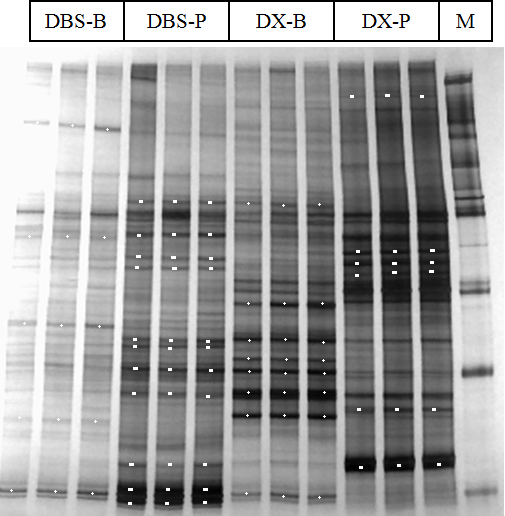

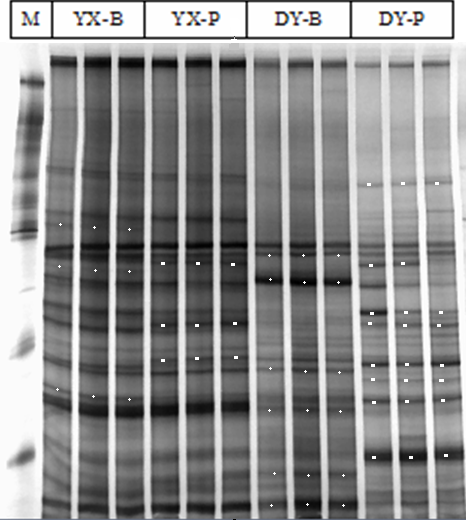


**A**


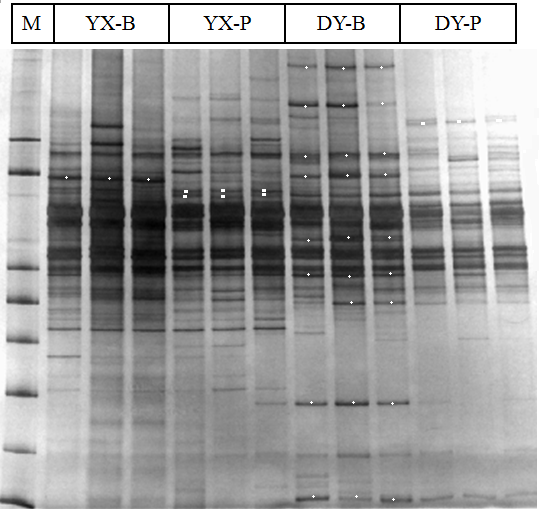

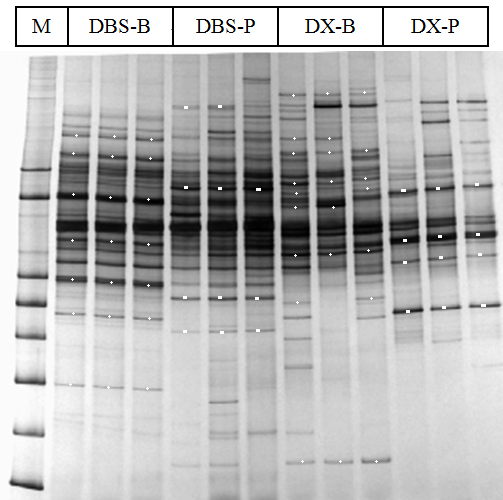


**B**

**Figure S2**

Supplement: Figure S2 — DGGE profiles of amplified bacterial 16S rRNA (A) and fungal 18S rRNA (B) gene fragments. YX-B and YX-P, Background and polluted soil of site YX; DX-B and DX-P, Background and polluted soil of site DX; DY-B and DY-P, Background and polluted soil of site DY; DBS-B and DBS-P, Background and polluted soil of site DBS, respectively. M, Marker. White asterisks highlight bands which intensified or only existed in the profiles of background soils; whereas, white rectangles highlight those which intensified or only existed in the polluted counterparts in a single site. (DOC) [file pone.0038858.s002.doc]
